# Early inflammatory mediator gene expression in two models of traumatic brain injury: *ex vivo* cortical slice in mice and *in vivo* cortical impact in piglets

**DOI:** 10.1186/s12974-015-0298-4

**Published:** 2015-04-18

**Authors:** David J Graber, Beth A Costine, William F Hickey

**Affiliations:** Department of Pathology, Geisel School of Medicine at Dartmouth, One Medical Center Drive, Lebanon, NH 03756 USA; Department of Neurosurgery, Massachusetts General Hospital and Harvard Medical School, 55 Fruit Street, Boston, MA 02114 USA

**Keywords:** Cytokines, Cerebral cortex, Chemokines, Injury, Mouse, Pig, Prostaglandin-endoperoxide synthase-2, Slice

## Abstract

**Background:**

The immunological response during the first 24 hours after traumatic brain injury (TBI) may be a critical therapeutic interval for limiting the secondary neuronal damage that is influenced by enhanced inflammatory mediator expression.

**Methods:**

To gain further insight of the early injury response, we examined the expression of several inflammatory genes by real-time qPCR as a function of time or distance from injury in two distinct mammalian models: an *ex vivo* mouse cortical slice injury system and an *in vivo* piglet model of brain injury.

**Results:**

Interleukin-1β (IL-1β), tumor necrosis factor-α (TNF-α), chemokine ligands 2 (CCL2), 3 (CCL3), 4 (CCL4), and prostaglandin-endoperoxide synthase 2 (PTGS2) mRNAs increased within 5 h after injury in mouse cortical slices. Chemokine and PTGS2 mRNAs remained elevated in slices at 24 h, whereas IL-1β and TNF-α expressions decreased from earlier peak levels. At 24 h after cortical injury in 1-month-old piglets, the expression of CCL2 mRNA was significantly increased in the lesion core and in the penumbra region. The expression of PTGS2, IL-1β, and TNF-α was variable among the piglets.

**Conclusions:**

These *in vitro* and large animal models of cortical injury expand our understanding of the early timing and spread of the immunological response and can serve as preclinical systems to facilitate the discovery of therapeutic agents for TBI aimed at regulating inflammatory mediator expression.

## Background

Traumatic brain injury (TBI) is a major health concern in contact sports and in the military and is the leading cause of death and disability in children and young adults. Though progress has been made in prevention and acute clinical management, a therapeutic that ameliorates the disability or mortality associated with TBI remains elusive. Despite the growing awareness of this public health problem, a greater translational effort in clinical trials for TBI is needed [1]. Expanding the preclinical research landscape might enhance our understanding of TBI pathophysiology and facilitate the discovery of therapeutics that improve recovery outcomes.

Secondary damage in surrounding brain tissue occurs following the primary insult. Pro-inflammatory mediator expression is enhanced soon after injury and is followed several days later by a period of enhanced expression of anti-inflammatory cytokines [2]. While the immune response in the days and weeks after TBI can benefit recovery by clearing cellular debris and producing neurotrophic factors, disproportionate expression of neurotoxic pro-inflammatory mediators in the first hours and days after TBI injury may be detrimental [3]. Thus, an excessive and/or persistent innate immune response emanating from the primary injury site may contribute to the severity of secondary damage.

The time course of inflammatory mediator expression is well-established in *in vivo* rodent models of TBI. Interleukin-1β (IL-1β) and tumor necrosis factor-α (TNF-α) increase within the first several hours after cortical injury but then return to baseline levels by 24 h [4-6]. Chemokine ligands 2 (CCL2, formerly known as monocyte chemoattractant protein-1), 3 (CCL3), 4 (CCL4), and prostaglandin-endoperoxide synthase 2 (PTGS2) increase early after injury and remain elevated for 24 h or longer [7-9]. IL-1β and TNF-α are cytokines associated with promoting further inflammation, vascular permeability, and neurotoxicity. Chemokines recruit inflammatory cells and enhance synthesis of other inflammatory mediators. PTGS2 converts arachidonic acid from the damaged cell membranes to prostaglandins that modulate local cerebral blood flow, recruit inflammatory cells, and enhance free radical synthesis. In these rodent models, enhancing cytokine levels or genetic removal alters post-TBI outcomes highlighting the importance of these mediators in recovery early after TBI [6,8,10].

While most of our understanding of the early expression and effects of inflammatory mediators in response to cortical injury is from the rodent models of TBI, the timing and contribution of long-term deficits in larger animal models and in human patients are less clear. An increase in TNF-α and IL-1β is detected in human cortical tissues early after injury [11]. An increase in CCL2 in human CSF after TBI is found early and remains elevated for over 12 h [8]. CCL3 and CCL4 in cerebral microdialysis fluid are also elevated for 24 h or longer [12]. It remains unclear whether regulating the synthesis of these pro-inflammatory mediators will be helpful. Treating human TBI with synthetic glucocorticoids, which inhibit the synthesis of many inflammatory mediators, has not provided favorable results [13]. Safer anti-inflammatory alternatives to glucocorticoids are being investigated and show promise [14].

Discovering new therapeutic agents with ideal immunomodulatory characteristics in the CNS may improve outcomes when administered early after TBI. This discovery effort will be facilitated by filling the gaps in preclinical translational research that exist between cell culture and rodent models and between rodent models and human studies. To fill these two gaps, we examine the early changes in the mRNA expression of inflammatory mediators following cortical injury in two understudied systems of TBI. In the first system, we use mouse cortical slices to evaluate the *ex vivo* changes in the mRNA expression of cytokines, chemokines, and PTGS2 that occur during the first 24 h after injury. Slices maintain neural cells in their normal microenvironment, and an acute innate immune response occurs following tissue sectioning. In the second system, we used piglets to assess the *in vivo* changes in the expression of these inflammatory genes 24 h after cortical impact at the lesion core and in the penumbra region. The piglet brain is gyrencephalic where the abundance of white matter, anatomy, vasculature, and pattern of development closely parallels humans [15]. These two systems of cortical injury both demonstrate increased expression of genes involved in the innate immunological response.

## Methods

### Mouse cortical slices

The Institutional Animal Care and Use Committee at Dartmouth College approved all mouse protocols. Adult (2 to 3 months old) female SJL/J mice (*n* = 8) were obtained from Jackson Laboratory (Bar Harbor, ME, USA) and housed for several weeks at the Borwell facility in Dartmouth’s Center for Comparative Medicine and Research. Acute cultures of cortical slices were prepared as described previously in more detail [16] with minor modifications. Brains were rapidly removed after carbon dioxide-inhaled euthanasia. Four to six slices of cortical tissue from each mouse brain were immediately transferred to RNAlater™ RNA Stabilization Reagent (Qiagen Sciences, Germantown, MD, USA) as a baseline (0 h) or submerged in 0.5 mL Dulbecco’s Modified Eagle Medium (Hyclone Laboratories, Logan, UT, USA) supplemented with fetal bovine serum (Hyclone; 10%) and glutamine (2 mM), penicillin (50 U/mL), and streptomycin (50 μg/mL) in a 48-well tissue culture tray. In contrast to studies utilizing slices for electrophysiology, sections are made without the neuroprotectant sucrose as a model of TBI [17]. Slices in media were incubated in 5% carbon dioxide and high humidity conditions for 0.5, 4.5, 6, or 24 h before being transferred to RNAlater^TM^ solution.

### Cortical injury in piglets

All protocols were in accordance with the guidelines of the American Veterinary Association and the National Institutes of Health (NIH) and were approved by the Animal Care and Use Committee of Massachusetts General Hospital. One-month-old male Yorkshire piglets (*n* = 6) were obtained from Earle Parsons & Sons, Inc. (Hadley, MA, USA) and were housed in the MGH Center for Comparative Medicine. Cortical impact has been described previously in more detail [18,19]. In brief, the piglets were anesthetized with ketamine and xyalzine and 5% isoflurane and maintained with 1% to 2% isoflurane. Atropine and buprenorphine were administered, and the pigs were intubated and mechanically ventilated. Core body temperature, end tidal CO_2_, and oxygen saturation were kept within a narrow range for the duration of the surgery. A craniectomy was performed at the junction of the right coronal and sagittal sutures, and the dura incised to expose the cortical surface. Cortical impact to the rostral gyrus was achieved via a spring-loaded device secured to the skull, and an indentor tip scaled to 1% of the volume of the brain was displaced over 4 msec. In injured animals, this produces a lesion size of 350 ± 50 mm^3^ or approximately 10% of the volume of the contralateral hemisphere 1 week after injury that typically extends down into the gyral white matter [19,20]. This age of piglet corresponds developmentally to human toddlers [15]. After injury, the dura was re-approximated, the skin was closed, and the piglets were recovered. Twenty-four hours after cortical impact, the piglets were deeply anesthetized and given buprenorphine, and the burr hole expanded from the previous day and a new burr hole placed contralateral to the injury and dura to access cortical tissue. The piglets were euthanized with an injection of Euthasol, and cortical samples (2 cm^3^) were obtained immediately from the lesion core, penumbra, 2 cm anterior to the injury, and corresponding cortical region from the contralateral hemisphere. The lesion core was the region directly under the indentor tip. The penumbra was the region surrounding the lesion core. Samples were divided and/or snap frozen on dry ice, stored at −80°C freezer, and then immersed in RNAlater^TM^ solution prior to RNA isolation.

### Real-time reverse transcriptase PCR

After removing excess RNAlater™ solution from the piglet or mouse cortical tissues, RNA was extracted using TRIzol Reagent (Life Technologies, Carlsbad, CA, USA). The eluted RNA was quantified by spectrophotometry, and 2 μg was reverse-transcribed using qScript cDNA SuperMix (Quanta Biosciences, Gaithersburg, MD, USA). Mouse oligonucleotide primer sets were described previously [16]. Piglet oligonucleotide primer sets, detailed in Table 1, were designed using NCBI/Primer-BLAST. Quantitative real-time PCR was performed using PerfeCTa SYBR Green FastMix with low ROX (Quanta Biosciences), 8 ng of mouse or piglet cortical cDNA, and 300 nM of a RT-PCR primer sets (IDT, San Jose, CA, USA). Settings for analysis using an ABI Fast 7500 machine were as follows: initial denaturation (95°C/3 min) was followed by 45 cycles of denaturation (95°C/1 s) and primer annealing (60°C/30 s). A melt curve was performed on all samples for quality control. Data was quantified by the 2^(−ΔΔCt)^ method using β-actin or glyceraldehyde 3-phosphate dehydrogenase (GAPDH) (EC 1.2.1.12) as internal control reference mRNAs [21]. In the mouse cortical slice experiment, mRNA from three slices collected at various incubation time points in two independent experiments (*n* = 6) was analyzed in triplicate and expressed as mean fold difference ± SEM relative to baseline levels (time 0) in a freshly isolated cortex. In the piglet cortical injury experiment (*n* = 6), cDNA from individual lesion core, penumbra, 2 cm anterior, and contralateral regions were analyzed in triplicate and expressed as average fold difference ± SEM relative to average levels in contralateral tissues. For analysis of mRNA changes in individual piglets (*n* = 6), CCL2 and PTGS2 (EC 1.14.99.1) mRNAs in penumbra and lesion core regions were expressed as fold difference relative to levels in the contralateral tissue from the same piglet.

**Table 1:** **Primer sequences for real-time qPCR analysis of piglet gene expression**

**mRNA**	**Swine primer sequence (5′ - 3′)**	**Amplicon** ^**a**^ **(bp)**	**Accession #** ^**b**^
IL-1β F^c^	AGGCACAAAGGCCATTCAGT	102	NM_001005149.1
IL-1β R^c^	ACTTCCTTGGCAGGTTCAGG		
TNF-α F	CATCTACCTGGGAGGGGTCT	109	NM_214022.1
TNF-α R	ACCTGCCCAGATTCAGCAAA		
CCL2 F	CACCAGCAGCAAGTGTCCTA	86	NM_214214.1
CCL2 R	CCCACTTCTGCTTGGGTTCT		
CCL3 F	TATTTTGAGACCAGCAGCCAGT	127	NM_001009579.1
CCL3 R	CATTCAGCTCCAGGTCAGAGAT		
CCL4 F	TTCACATACACCGTGCGGAA	148	NM_213779.1
CCL4 R	ACTCCTGGACCCAGTCATCA		
PTGS2 F	CCAGCACTTCACCCATCAGT	132	NM_214321.1
PTGS2 R	AGGCGCAGTTTATGCTGTCT		
β-actin F	CAAGCAGGAGTACGACGAGT	145	XM_003357928.2
β-actin R	GGCTGGCATGAGGTGTGTA		
GAPDH F	AAGGTCGGAGTGAACGGATT	149	NM_001206359.1
GAPDH R	CCGTGGGTGGAATCATACTGG		

^**a**^Predicted number of base pairs (bp) in PCR product; ^b^NCBI reference sequence; ^c^F, forward primer; R, reverse primer. CCL2, chemokine ligand 2; CCL3, chemokine ligand 3; CCL4, chemokine ligand 4; GAPDH, glyceraldehyde 3-phosphate dehydrogenase; IL-1β, interleukin-1β; PTGS2, prostaglandin-endoperoxide synthase 2; TNF-α, tumor necrosis factor-α.

### Statistics

GraphPad Prism Software (La Jolla, CA, USA) was used to generate graphs and conduct statistical analysis. One-way ANOVA followed by Dunnett’s multiple comparison test was used for comparisons of various incubation times relative to baseline amounts in mouse slices. Student’s *t* test was used for comparisons of ipsilateral cortical regions relative to uninjured contralateral regions in the piglets. The correlation between the mRNA fold changes among individual piglets was analyzed by a linear regression model. Statistical significance was defined as *P* < 0.05.

## Results

### Inflammatory gene expression in mouse cortical slices over time

**Figure 1:**
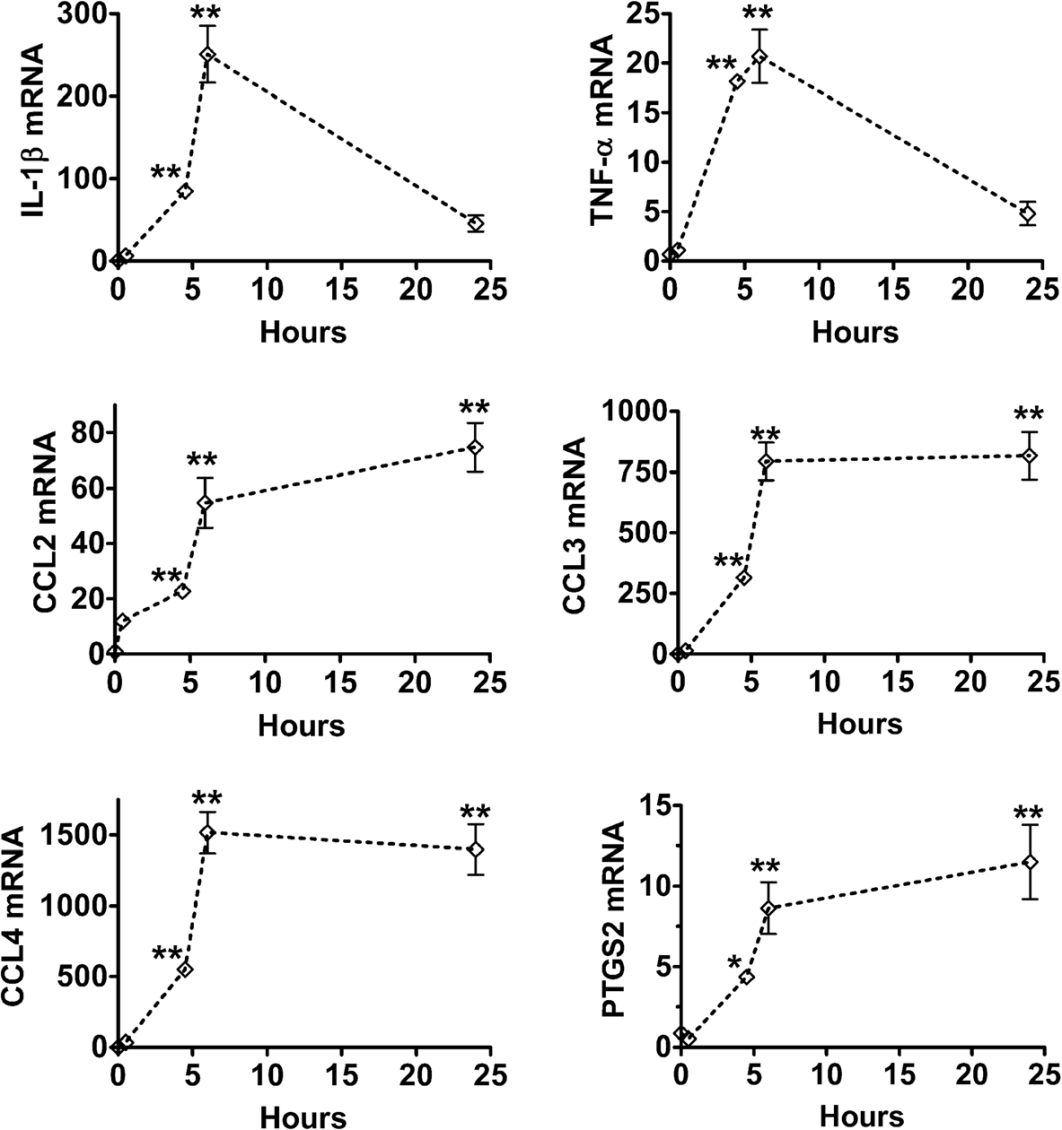
Expression of inflammatory genes in mouse cortical slices incubated on various times. IL-1β, TNF-α, CCL2, CCL3, CCL4, and PTGS2 mRNAs were measured at 0, 0.5, 4.5, 6, and 24 h in culture. Target mRNAs were normalized to β-actin mRNA as an endogenous control reference, and differences among the groups were tested with Dunnett’s multiple comparison test. *n* = 6 per time point. *Means ± SEM differ *P* < 0.05 relative to time zero; **means ± SEM differ *P* < 0.01 relative to time zero. CCL2, chemokine ligand 2; CCL3, chemokine ligand 3; CCL4, chemokine ligand 4; GAPDH, glyceraldehyde 3-phosphate dehydrogenase; IL-1β, interleukin-1β; PTGS2, prostaglandin-endoperoxide synthase 2; TNF-α, tumor necrosis factor-α.

To analyze the transcriptional changes in cortical slices, abundance of mRNA was measured in slices incubated for 0.5, 4.5, 6, and 24 h. Freshly isolated cerebral cortices were used as the 0 h baseline values. IL-1β and TNF-α transcripts increased from 0 to 6 h and then decreased between 6 and 24 h (Figure 1). CCL2-4 and PTGS2 mRNAs increased from 0 to 6 h and remained elevated at 24 h (Figure 1).

### Regional inflammatory gene expression in piglets after cortical impact, *in vivo*

**Figure 2:**
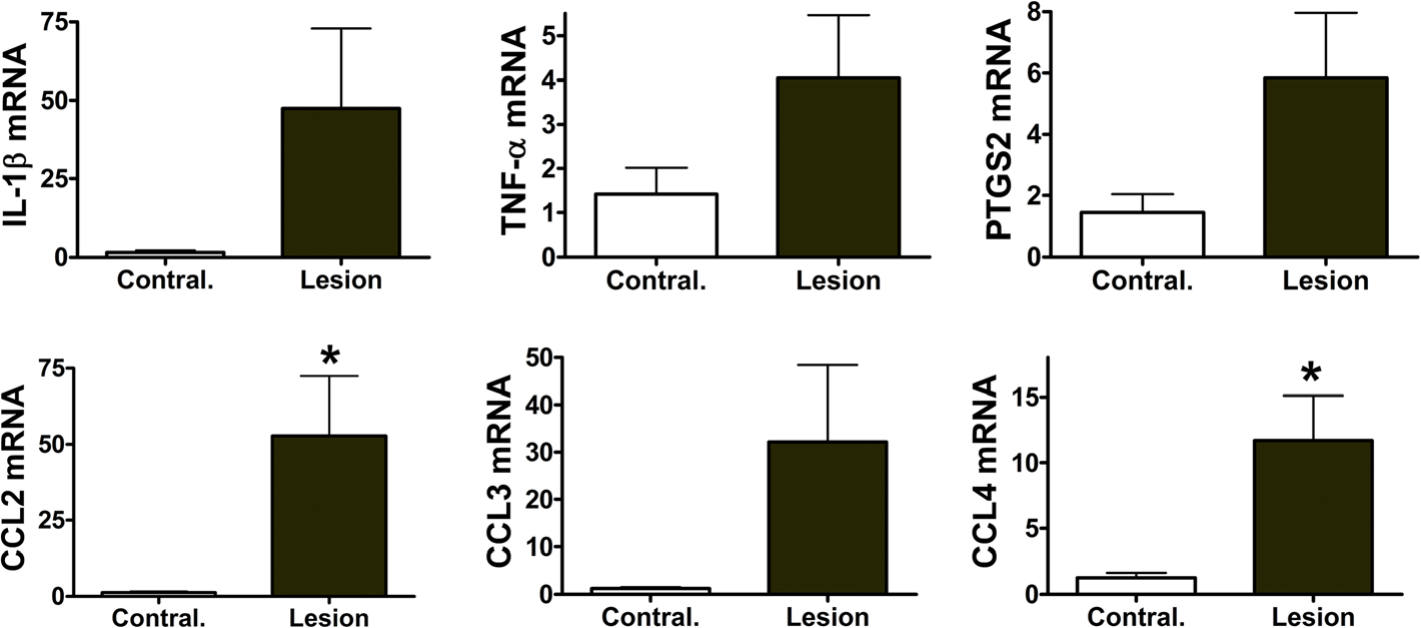
Expression of inflammatory genes in piglet cerebral cortex lesion 24 hours after cortical impact. IL-1β, TNF-α, CCL2, CCL3, CCL4, and PTGS2 mRNAs in the lesion core were expressed as mean fold change (±SEM) relative to mRNA abundance in cortical tissues from a similar region in the uninjured contralateral hemisphere. Target mRNAs were normalized to GAPDH mRNA as an endogenous control reference, and differences among the groups were tested with Student’s *t* test. *Means ± SEM differ *P* < 0.05 relative to contralateral region (*n* = 6). CCL2, chemokine ligand 2; CCL3, chemokine ligand 3; CCL4, chemokine ligand 4; IL-1β, interleukin-1β; PTGS2, prostaglandin-endoperoxide synthase 2; TNF-α, tumor necrosis factor-α.

**Figure 3:**
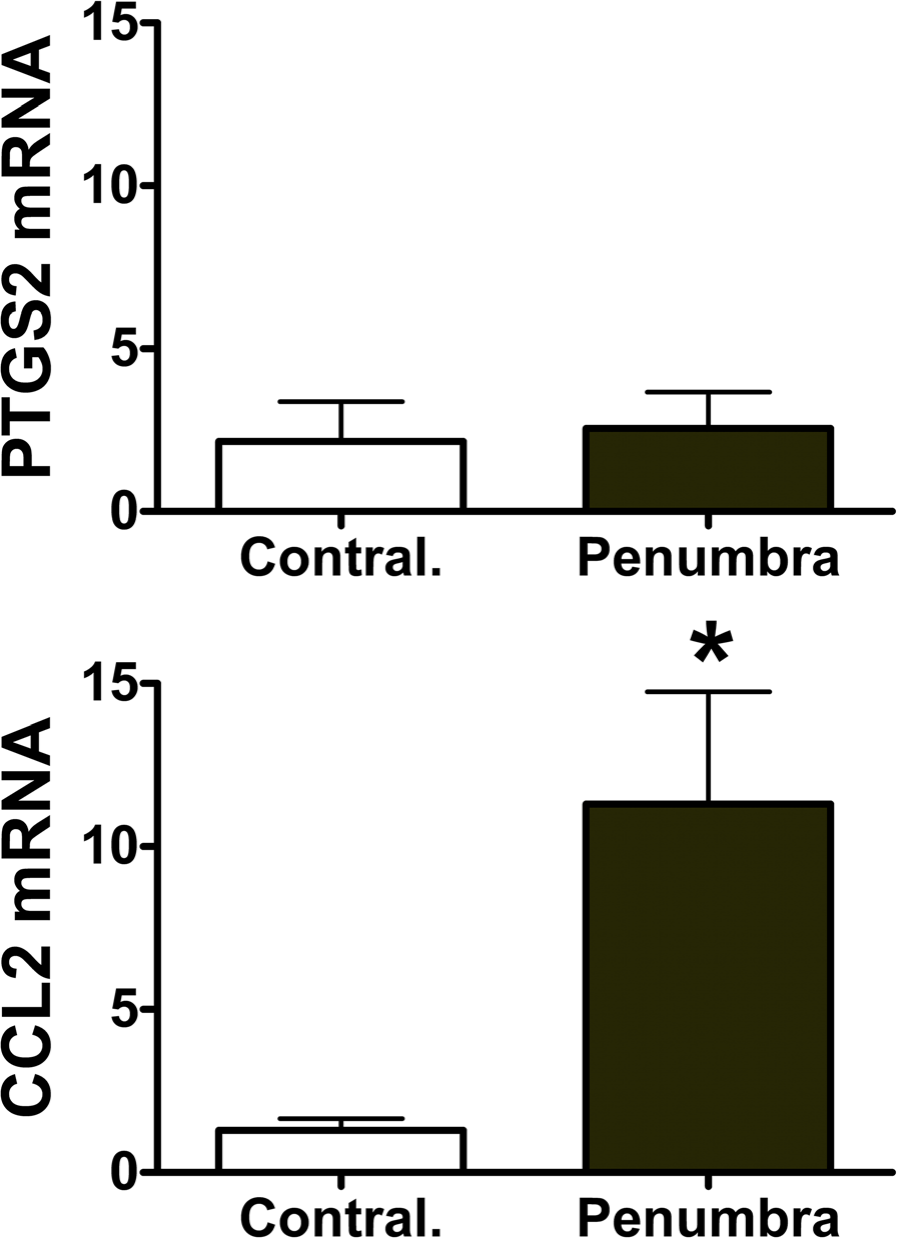
Expression of PTGS2 and CCL2 in the penumbra region of piglet cerebral cortex 24 hours after cortical impact. Messenger RNAs in the adjacent lesion core in the penumbra were expressed as mean fold change (±SEM) relative to mRNA abundance in cortical tissues from a similar region in the uninjured contralateral hemisphere. Target mRNAs were normalized to GAPDH mRNA as an endogenous control reference and differences among groups tested with Student’s *t* test. *Means ± SEM differ *P* < 0.05 relative to contralateral region (*n* = 6). CCL2, chemokine ligand 2; PTGS2, prostaglandin-endoperoxide synthase 2.

We analyzed next the level of inflammatory transcripts in piglet cerebral cortices after a cortical impact. Tissues from lesion core, penumbra, and 2-cm anterior to the penumbra were collected 24 h after injury for comparison to cortical tissue collected from the corresponding region on the uninjured contralateral side. While GAPDH and β-actin are both stable endogenous reference genes in uninjured pig cerebral cortex [22], we found GAPDH (mean critical threshold values across four cortical regions after injury ± SEM; 19.4 ± 0.1) to be more stable than β-actin (16.8 ± 0.2) following cortical injury. GAPDH mRNA was used as an endogenous reference for piglet tissues. The expressions of CCL2 and CCL4 mRNAs increased significantly in the lesion core tissues 24 h after injury (Figure 2). Average levels of IL-1β, TNF-α, PTGS2, and CCL3 mRNAs appeared elevated, but there was a high degree of variability and these changes were not statistically significant. CCL2 mRNA also increased in the penumbra region (Figure 3). No change was found in cortical tissue adjacent to the penumbra at 2-cm anterior to the injury in the same hemisphere vs. contralateral hemisphere (data not shown).

**Figure 4:**
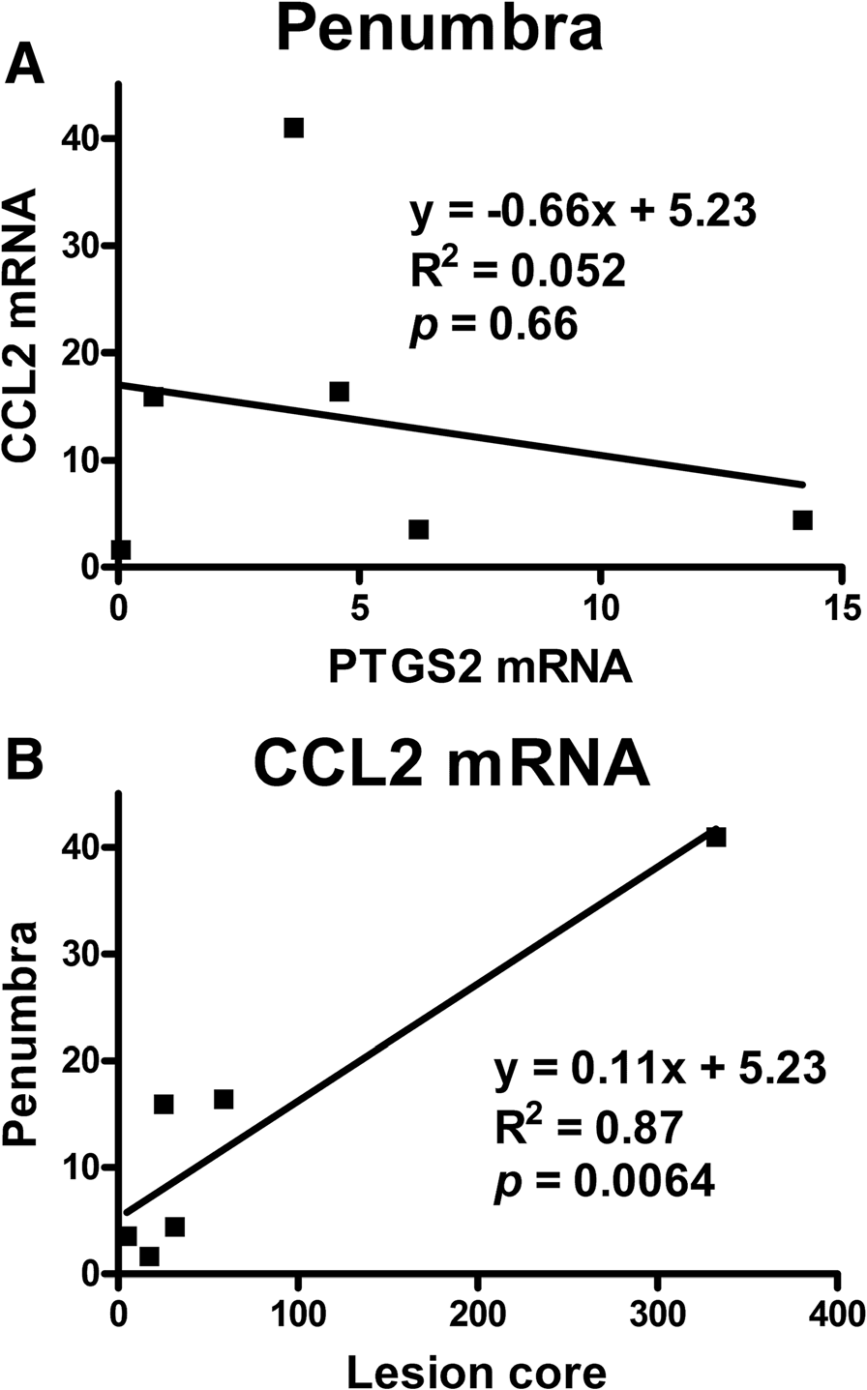
Correlation of CCL2 mRNA changes in piglet cerebral cortex 24 hours after cortical impact. Expressions of mRNA were normalized to GAPDH mRNA and expressed as mean fold change relative to mRNA expressed in contralateral tissue from the same piglet. **(A)** In the penumbra, changes in CCL2 mRNA were independent to changes in PTGS2 mRNA. **(B)** CCL2 mRNA changes in the penumbra correlated to CCL2 mRNA changes in the lesion core. CCL2, chemokine ligand 2.

Some genes are found to be less stable across individual outbred pigs even in uninjured cerebral cortex [22]. Therefore, we examined the distribution of the PTGS2 and CCL2 mRNA expressions in cerebral cortex in individual piglets. As shown in Table 2, the critical threshold values in the contralateral hemisphere for CCL2 (range 4.2 to 6.9) reveal less mRNA variability among the piglets in comparison to PTGS2 (4.0 to 9.8). CCL2 mRNA increased in the penumbra relative to contralateral tissue levels within the same piglet, whereas changes in PTGS2 were either increased or decreased in different piglets. No linear correlation between the changes of CCL2 and PTGS2 mRNAs was found in the penumbra, but a strong correlation between CCL2 mRNA changes in penumbra and lesion core was observed (Figure 4).

**Table 2:** **Abundance of mRNAs in cerebral cortex among individual piglets 24 h after injury**

	**CCL2**			**PTGS2**		
	**Ct**_**CCL2**_ **-Ct**_**GAPDH**_^**a**^		**2** ^**−ΔΔCt**^	**Ct**_**PTGS2**_ **-Ct**_**GAPDH**_^**a**^		**2** ^**−ΔΔCt**^
**Piglet**	**Contralateral**	**Penumbra**	**Fold change** ^**b**^	**Contralateral**	**Penumbra**	**Fold change** ^**b**^
#1	6.01	5.30	1.64	4.00	8.15	0.056
#2	6.48	2.49	15.92	4.97	5.43	0.73
#3	6.91	1.56	40.98	7.96	6.09	3.63
#4	4.38	2.24	4.40	7.77	3.94	14.19
#5	4.80	0.77	16.40	8.51	6.32	4.58
#6	4.21	2.38	3.55	9.79	7.15	6.23

^a^Ct = mean critical thresholds of target minus internal control reference; ^b^fold change in penumbra relative to contralateral in individual piglets. CCL2, chemokine ligand 2; GAPDH, glyceraldehyde 3-phosphate dehydrogenase; PTGS2, prostaglandin-endoperoxide synthase 2.

## Discussion

The immunological response to neural injury is predominantly investigated in the *in vivo* rodent models. Alternatively, glia are activated *in vitro* with endotoxins or cytokines in dissociated cell culture systems. Yet, therapies identified in current model systems have not easily translated to clinically effective treatments [23]. Additional preclinical systems will expand our understanding of the acute inflammatory response to cortical injury and may facilitate the discovery of pharmacological agents that attenuate secondary damage. In this study, we report the transcriptional changes of inflammatory genes in two cortical injury systems that can help fill the preclinical translational gaps in knowledge that exist before and after *in vivo* rodent models.

Microglia are the resident CNS cells derived from monocyte/macrophage lineage and are the early responders to neural injuries. While microglia can be isolated or immortalized for use in highly valuable cell culture studies, one disadvantage to this system is that dissociated microglia are separated from their normal neural contacts. Microglial phenotype and function are regulated by the unique and complex cellular and extracellular signaling molecules [24]. Studying slices of CNS tissue *ex vivo* has the advantage of maintaining glia in their neural microenvironment, and the sectioning of tissue causes an immunological response without the requirement of endotoxins. While the change in acute expression of inflammatory mediators in spinal cord slices reveals a similar profile to observations in spinal cord injury in the rodents *in vivo* [25,26], it is not known whether slices of cerebral cortex will demonstrate a similar pattern to *in vivo* models of TBI. The IL-1β and TNF-α expressions increase in the cortex within the first few hours in the rodent models of TBI before returning to baseline levels by 24 h [4,5]. Similarly, the expression of these cytokines increased in the first 6 h and then decreased by 24 h in mouse cortical slices. In the *in vivo* rodent models, the expression of chemokines and PTGS2 increases within hours of cortical injury and, unlike IL-1β and TNF-α, remains at elevated levels at 24 h or beyond [7-9]. Remarkably, these particular transcripts increased early and remained elevated at 24 h in cortical slices. These findings suggest that cells resident to the CNS are largely responsible for the acute immunological response to injury because the *ex vivo* slice system has negligible infiltration of leukocytes. The inflammatory gene expression in slices is regulated by a genetic background [16] and can be modified by pharmacological agents in the explant medium [25-27]. The slice system of cortical injury represents an uncomplicated method to investigate the early immunological response and is amendable to transgenic experiments and for screening of active anti-inflammatory agents that target inflammatory expression at the level of transcription. Future studies should examine whether expression of cytokines and chemokines is increased at the protein level, which will enhance the utility of the cortical slice injury system.

While cortical slices help fill the translational gap between cell culture and intact rodent models of injury, a gap in knowledge of acute cortical injury response also exists between *in vivo* rodent models and clinical studies in humans. Swine have gyrencephalic brains similar to humans in which 50% of the brain is comprised of white matter in contrast to the lissencephalic rodent brain that contains 15% white matter. Outbred swine, which are less expensive than primate species and physiologically similar to humans, are increasingly being used to study other trauma and other diseases involving the immune system such as organ transplant biology as they have fewer immunologic hurdles to overcome from swine to human [28]. Swine are increasingly being utilized as models for pediatric traumatic brain injury [18,29] and blast injuries in the military [30]. Swine are particularly useful in understanding TBI in children as the timing of the peak growth spurt in the piglet occurs around the time of birth in humans but occurs prior to birth in primates [15]. TBI superimposed upon development adds complexity to the problem of therapeutics as therapies may have the opposite effect in the immature brain compared to the mature brain and may interfere with normal development to a greater degree of any benefit in treating the brain injury [29].

This report demonstrates the early transcriptional inflammatory response following cortical injury in piglets. In agreement with previous findings using outbred swine [22], which parallel genetic diversity in humans, we found a high degree of variability in mRNA expression for some genes. PTGS2 mRNA in the cerebral cortex was particularly inconsistent in piglets. IL-1β and TNF-α mRNA levels were also variable in the lesion core 24 h after injury. Since these cytokines increase in the early hours after injury in the rodent models of TBI before returning to baseline at 24 h [4,5], future studies should evaluate IL-1β and TNF-α expressions at earlier time periods after injury in piglets. Chemokines, on the other hand, were more stable and represent reliable markers of acute inflammatory changes in response to injury in piglets. Increased CCL2 mRNA was found in the lesion core 24 h after injury. An increase in CCL2 was also found in the penumbra region, which revealed a positive linear correlation to changes in the lesion core within piglets. CCL2, as opposed to PTGS2, may represent a more reliable biomarker for injury severity and for drug discovery efforts aimed at regulating the immunological reaction. Insight into stable and inconsistent inflammatory transcripts in response to TBI in outbred swine may help in identifying reliable biomarkers useful in the development of therapies to overcome the inherent variability in humans. Here, we initiate study into the inflammatory response in immature swine and plan on determining the age-dependent differences in the acute injury response, which may explain the previously described age-dependent lesion size among stages of immaturity and reveal age-specific inflammatory targets [18,19]. Here, we have used injured piglets as their own control comparing cortical tissue from the ipsilateral vs. contralateral tissue similar to our previous studies [18,29]; however, immune modulators may indeed be elevated in the contralateral hemisphere thus blunting our observed increases and perhaps contributing to the variability among subjects in the contralateral cortical tissue. Future studies should include sham piglets to determine if the inflammatory modulators are increased in the cortex contralateral to the impact in this immature, large animal model of TBI.

New experimental systems to investigate the acute immunological reaction may facilitate the discovery of early interventional drugs to mitigate the disability and fatality after TBI. In the current study, two disparate mammalian systems of cortical injury illustrate an early increase in pro-inflammatory transcripts. The mouse cortical slice system reveals a temporal change in cytokine and chemokine expressions that is similar to the *in vivo* changes reported with the rodent models of TBI. This slice injury approach can bridge the gap in experimental systems that exists between higher throughput cell culture and lower throughput animal models to facilitate immunotherapeutic discovery and the understanding of the acute immunological reaction to cortical injury. Later stages of preclinical development of immunomodulatory drugs would benefit from injury models using swine, which may more reliably model the brain anatomy and subject variability in human studies. The *ex vivo* mouse cortical slice injury system and the *in vivo* piglet model of brain injury can serve as additional preclinical systems to facilitate the discovery of therapeutic agents for TBI aimed at regulating early inflammatory mediator expression.

## Abbreviations

CCL2: chemokine ligand 2
CCL3: chemokine ligand 2
CCL4: chemokine ligand 3
IL-1β: interleukin-1β
PTGS2: prostaglandin-endoperoxide synthase 2
TBI: traumatic brain injury
TNF-α: tumor necrosis factor-α
